# Arthralgia among Patients with COVID-19 Infection Admitted to the Department of Medicine of a Tertiary Care centre: A Descriptive Cross-sectional Study

**DOI:** 10.31729/jnma.8155

**Published:** 2023-05-31

**Authors:** Ujjwol Prasad Risal, Mrikchhya Ghimire, Asmita Karki, Nahakul Shahi, Suravi Pandey

**Affiliations:** 1 Department of Internal Medicine, Hospital for Advanced Medicine and Surgery, Dhumbarahi, Kathmandu, Nepal; 2 Department of General Practice and Emergency Medicine, BP Koirala Institute of Health Sciences, Ghopa, Dharan, Nepal; 3 Research Unit, Hospital for Advanced" Medicine and Surgery, Dhumbarahi, Kathmandu, Nepal; 4 Central Jail Hospital, Tripureshwor, Kathmandu, Nepal

**Keywords:** *arthralgia*, *COVID-19*, *prevalence*, *tertiary care*

## Abstract

**Introduction:**

COVID-19 is a global pandemic that has affected millions of people worldwide. It predominantly affects the respiratory tract causing various respiratory symptoms. It also causes various musculoskeletal symptoms in the form of arthralgia and myalgia which may be incapacitating for some patients. The objective of this study was to find out the prevalence of arthralgia among patients with COVID-19 infection admitted to the Department of Medicine.

**Methods:**

This descriptive cross-sectional study was carried out in the Department of Internal Medicine of a tertiary care centre. Data from March 2020 to May 2021 were collected between 2 December 2021 and 20 December 2021 from the hospital records. Ethical approval was obtained from the Ethical Review Board (Reference number: 1312). All patients who were admitted with the diagnosis of COVID-19 infection based on positive Reverse Transcriptase-Polymerase Chain Reaction for COVID-19 were included in the study. Convenience sampling method was used. Point estimate and 95% confidence interval were calculated.

**Results:**

Among 929 patients included in the study, the prevalence of arthralgia was found to be 106 (11.41%) (10.30-12.51, 95% Confidence Interval). The mean age of these patients was 52.81±17.46 years.

**Conclusions:**

The prevalence of arthralgia in COVID-19-infected patients was similar to other similar studies done in similar settings.

## INTRODUCTION

COVID-19 is a global pandemic that has affected millions of people worldwide since its emergence in Wuhan, China in December 2019.^1^ It primarily affects the respiratory tract giving rise to symptoms of cough, fever, rhinorrhea, sore throat and difficulty breathing.^2^ Involvement of other systems such as the cardiovascular, nervous and gastrointestinal tract is also common with COVID-19 infection giving rise to various organ-specific symptoms.^3-5^

COVID-19 infection has also been found to cause myalgia, arthralgia, arthritis, backache and fatigue, particularly in the prodromal stage.^5^ These symptoms are similar to the ones seen in various rheumatological disorders. The frequency of the above manifestations is different in different studies.^6^ However no study has been done from Nepal to date that primarily looks at the frequency of arthralgia in COVID-19 infection.

The objective of this study was to find out the prevalence of arthralgia among patients with COVID-19 infection admitted to the Department of Medicine.

## METHODS

A descriptive cross-sectional study was carried out in the Department of Internal Medicine at the Hospital for Advanced Medicine and Surgery (HAMS), Dhumbarahi, Kathmandu, Nepal. Ethical approval was obtained from Ethical Review Board (Reference number: 1312). Data from March 2020 to May 2021 were collected between 2 December 2021 to 20 December 2021 from the hospital records. All patients who were admitted to the Department of Internal Medicine of HAMS with the diagnosis of COVID-19 infection with complete hospital data based on positive Reverse Transcriptase-Polymerase Chain Reaction (RT-PCR) for COVID-19 were included in the study. Patients who were less than 12 years of age, who already had an underlying rheumatological disease and who had insufficient records were excluded from the study. Convenience sampling method was used. The sample size was calculated using the following formula:


$$\begin{array}{ll} \text{n} & ={\text{Z}}^{2}\times \frac{\text{p}\times \text{q}}{{\text{e}}^{\text{2}}}\\ & =1.96^{2}\times \frac{0.50\times 0.50}{0.05^{2}}\\ & =385 \end{array}$$

Where,

n = minimum required sample size
Z = 1.96 at 95% Confidence Interval (CI)
p = prevalence taken as 50% for maximum sample size calculation
q = 1-p
e = margin of error, 5%

The minimum required sample size obtained was 385. However, a sample size of 929 was taken for the study. The following definitions were used for the study.

Arthralgia: Patients describing pain in one or more joints without objective signs of inflammation.^7^ Myalgia: Patients describing pain in one or more groups of muscles without objective signs of weakness or laboratory investigations suggestive of myositis.^7^ Leukopenia: Defined as white blood cell (WBC) count less than 4000/mm.^3,8^ Thrombocytopenia: Defined as platelet count less than 150,000/mm.^3,8^

Data were entered in Microsoft Excel 2016 and analysis was done using IBM Statistics SPSS 23.0. Point estimate and 95% CI were calculated.

## RESULTS

**Figure 1:**
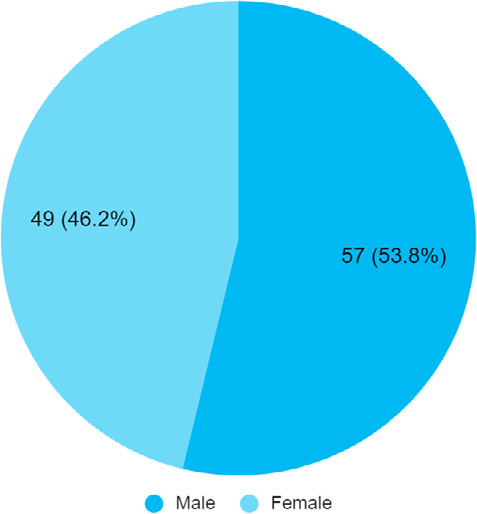
Gender-wise distribution of patients with arthralgia (n= 106).

Among 929 patients with COVID-19, the prevalence of arthralgia was 106 (11.41%) (10.30-12.31, 95% CI). The mean age was 52.81±17.46 years (Figure 1).

**Figure 2:**
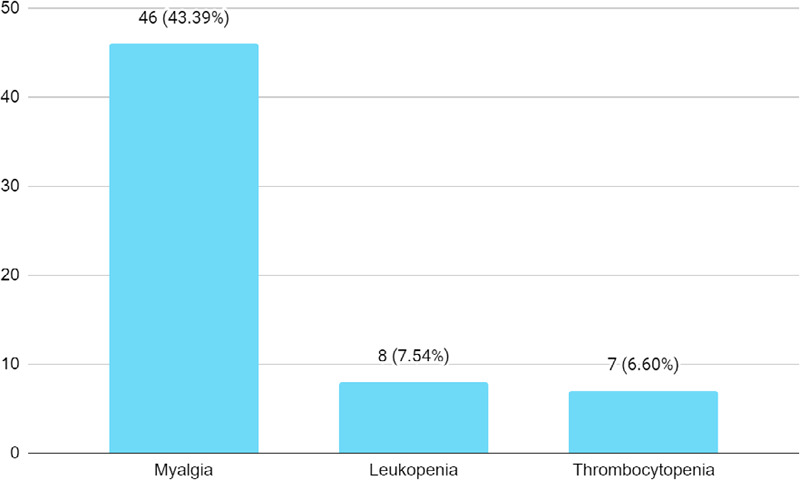
Other manifestations in patients with arthralgia (n= 106).

Among patients with arthralgia, myalgia was seen in 46 patients (43.39%) (Figure 2).

## DISCUSSION

In this study, the prevalence of arthralgia was found to be 11.4% among all hospitalized COVID-19 patients. This finding is very similar to the findings from other similar studies which had shown the prevalence of arthralgia to be 10-15%.^9^ However in another hospital-based study of 294 patients, 30% reported musculoskeletal complaints, among which 5.7% had arthralgia.^10^ This difference could be due to the fact that the population in the above study was predominantly younger with a median age of 36 years and with a significantly higher number of males whereas our patient population was predominantly older with almost equal distribution of sex which might have resulted in such a difference. In another study done on 417 COVID-19 patients in 12 hospitals in Europe, arthralgia was found in 31% of patients.^11^ This difference in finding may be due to the fact that the study involved only European patients with predominantly older age groups which could have caused such a difference. The mechanism of arthralgia in COVID-19 infection is currently unknown. Direct entry into synovial tissue via angiotensin converting enzyme-2 (ACE-2) receptor, immune complex deposition, transient synovitis and enthesopathy are some of the hypotheses proposed in causing arthralgia.^10,12^

Myalgia was seen in 43.39% of patients with arthralgia in this study. Various studies have shown that COVID-19 infection causes myalgia in a significant proportion of affected patients. In one report, myalgia was reported in 36% of COVID-19 patients, especially in early disease.^13^

The exact mechanism of myalgia is still unknown but various hypotheses have been proposed for its causation. One mechanism could be the direct entry of the virus through the ACE-2 receptor and subsequent muscle damage.^14^ Another mechanism could be related to the spinal ACE2/Ang (1-7)/Mas receptor pathway.^15^ Yet another hypothesis has proposed the role of interleukin-6 (IL-6) which in turn induces prostaglandin E2 production which results in myalgia.^16^ Whatever may be the mechanism, myalgia has been found to be a prominent and early symptom of COVID-19 infection.

As far as laboratory findings were concerned, leukopenia and thrombocytopenia were seen in 7.54 % and 6.60 % of patients with arthralgia. In a survey done on 1099 patients with COVID-19, leukopenia was seen in 33.70% of patients and thrombocytopenia in 36.2%^8^. However, no study has looked at the prevalence of leukopenia and thrombocytopenia in COVID-19 patients with arthralgia and its correlation which needs further studies.

The strength of our study is that it is the first of its kind in Nepal that has specifically looked at the frequency of arthralgia in COVID-19 patients. Our study is one of the largest studies in terms of sample size. Our study has certain limitations too. It was a retrospective study which could have resulted in bias regarding the specific rheumatological symptoms of the patient as the treating physicians in the clinics focus on the more serious symptoms like fever, cough, shortness of breath and chest pain. Therefore, many rheumatological symptoms might not have been recorded in the patient's file. Also, it was a hospital-based study, which might have caused bias in recording the symptoms as more serious patients were only admitted to the hospital. Therefore, this finding could not be extrapolated to the whole population. Another limitation was the strain of the COVID-19 virus was not characterized since different strains have been found to have different clinical and laboratory manifestations.

## CONCLUSIONS

The prevalence of arthralgia in admitted COVID-19 patients was similar to other studies done in similar settings. Myalgia was found to be a common symptom associated with arthralgia. Further large-scale studies need to be done for proper correlation of arthralgia and other clinical and laboratory manifestations.
